# *Burkholderia pseudomallei* Colony Morphotypes Show a Synchronized Metabolic Pattern after Acute Infection

**DOI:** 10.1371/journal.pntd.0004483

**Published:** 2016-03-04

**Authors:** Philipp Gierok, Christian Kohler, Ivo Steinmetz, Michael Lalk

**Affiliations:** 1 Institute of Biochemistry, Ernst-Moritz-Arndt-University, Greifswald, Germany; 2 Friedrich Loeffler Institute of Medical Microbiology, University Medicine Greifswald, Greifswald, Germany; University of Tennessee, UNITED STATES

## Abstract

**Background:**

*Burkholderia pseudomallei* is a water and soil bacterium and the causative agent of melioidosis. A characteristic feature of this bacterium is the formation of different colony morphologies which can be isolated from environmental samples as well as from clinical samples, but can also be induced *in vitro*. Previous studies indicate that morphotypes can differ in a number of characteristics such as resistance to oxidative stress, cellular adhesion and intracellular replication. Yet the metabolic features of *B*. *pseudomallei* and its different morphotypes have not been examined in detail so far. Therefore, this study aimed to characterize the exometabolome of *B*. *pseudomallei* morphotypes and the impact of acute infection on their metabolic characteristics.

**Methods and Principal Findings:**

We applied nuclear magnetic resonance spectroscopy (^1^H-NMR) in a metabolic footprint approach to compare nutrition uptake and metabolite secretion of starvation induced morphotypes of the *B*. *pseudomallei* strains K96243 and E8. We observed gluconate production and uptake in all morphotype cultures. Our study also revealed that among all morphotypes amino acids could be classified with regard to their fast and slow consumption. In addition to these shared metabolic features, the morphotypes varied highly in amino acid uptake profiles, secretion of branched chain amino acid metabolites and carbon utilization. After intracellular passage *in vitro* or murine acute infection *in vivo*, we observed a switch of the various morphotypes towards a single morphotype and a synchronization of nutrient uptake and metabolite secretion.

**Conclusion:**

To our knowledge, this study provides first insights into the basic metabolism of *B*. *pseudomallei* and its colony morphotypes. Furthermore, our data suggest, that acute infection leads to the synchronization of *B*. *pseudomallei* colony morphology and metabolism through yet unknown host signals and bacterial mechanisms.

## Author Summary

Melioidosis is a common disease in Northern Australia and East Asia, with regional mortality rates of up to 40%. Clinical manifestations range from soft tissue infections to severe sepsis. It is caused by the Gram negative saprophytic water and soil bacterium *Burkholderia pseudomallei*, which forms a variety of colony morphologies on solid agar. Various morphotypes appear after the bacterium is exposed to physiological stress conditions or underwent the process of infection, yet the physiological function is unclear. Metabolism is closely linked to virulence in many pathogens, and since metabolic data are not available so far for this bacterium, we monitored the nutrition uptake and metabolite secretion of *B*. *pseudomallei* morphotypes. Interestingly, despite typical genes responsible for gluconate production are missing in the *B*. *pseudomallei* genome, we observed high amounts of gluconate in the extracellular space. Furthermore, we were able to investigate metabolic differences among the morphotypes and identified synchronization in morphology and metabolism after infection as an adaptation to the host environment.

## Introduction

Colony morphology variants are described for many bacterial pathogens that are able to induce pneumonia including *Pseudomonas aeroginosa* [1] *Staphylococcus aureus* [2] and *Burkholderia cepacia* complex [3]. It is also a long known phenomenon of the Gram negative water and soil proteobacterium *Burkholderia pseudomallei*, which is the causative agent of melioidosis [4,5]. This disease occurs predominantly in Northern Australia, Southeast Asia, China and Taiwan but additional cases and environmental isolates of *B*. *pseudomallei* have been reported from several regions between latitude 20°N and 20°S [6–8]. Clinical manifestations are highly diverse including soft tissue lesions, abscess formation, sepsis and pneumonia, with the latter being the most frequent clinical presentation of this disease [4,8]. The isolation of isogenic *B*. *pseudomallei* morphotypes out of patients indicates a significant role for these morphotypes in adaptation during human melioidosis [9]. *In vitro* studies have shown that the appearance of various *B*. *pseudomallei* morphotypes can be linked to starvation stress, iron limitation, growth temperature and presence of antibiotics [9]. However a clear connection between morphotype formation and metabolism as described for *S*. *aureus* or *Pseudomonas fluorescence* has not been established so far [10–12]. Functional studies indicate that infection relevant parameters like adhesion and intracellular replication differ between the morphotypes [9,13]. Additionally, a higher susceptibility to reactive oxygen species, overexpression of the arginine deaminase system and flagellin was observed [13,14]. However, the question, if morphotype formation affects the bacterial metabolism should be addressed, since in many intracellular pathogens metabolism is closely connected to virulence and depends on host derived nutrients (reviewed in [15,16]). Metabolic adaptation of *B*. *pseudomallei* to the host environment includes the expression of metabolic genes for alternative carbon sources and the downregulation of TCA-cycle, glycolysis and oxidative phosphorylation [17,18]. Yet gene expression data only can indicate metabolic activities and to our knowledge actual metabolic investigations have not been carried out on *B*. *pseudomallei* so far. We therefore aimed in this study to gain a deeper insight into the diversity of colony morphology variants of *B*. *pseudomallei* on a metabolic level. Thus we investigated the uptake of amino acids, glucose and other carbon sources as well as the secretion of metabolites into the extracellular space. Thereby, we found so far unknown shared metabolic characteristics of *B*. *pseudomallei* morphotypes and morphotype specific secretion patterns. Our intention was also to address the question whether these metabolic features are affected by the process of infection in a murine macrophage cell culture model and in an acute pneumonia mouse model. Altogether our data indicate that, despite some variation, metabolic principles are shared among *B*. *pseudomallei* colony morphology variants and that the acute infection event synchronizes colony morphology as well as metabolism.

## Materials and Methods

### Ethics statement

All the animal experiments described in the present study were conducted in strict accordance with the recommendations in the Guide for the Care and Use of Laboratory Animals of the National Institutes of Health. All animal studies were conducted under a protocol approved by the Landesamt für Landwirtschaft, Lebensmittelsicherheit und Fischerei Mecklenburg-Vorpommern (LALLF M-V; 7221.3–1.1-020/11). All efforts were made to minimize suffering and ensure the highest ethical and human standards.

### Bacterial strains

All experiments with *B*. *pseudomallei* were carried out in biosafety level 3 (BSL3) laboratories. Two different *B*. *pseudomallei* strains were used in this study. The sequenced *B*. *pseudomallei* strain K96243 was originally isolated in 1996 from a 34-year-old female diabetic patient in Thailand [19] and has been used as a reference strain in many studies [20–22]. The second strain is the yet unsequenced *B*. *pseudomallei* strain E8, an environmental isolate from Ubon Ratchathani, Thailand [23,24].

### Isolation of colony morphotypes after nutrient starvation stress

Both strains were cultivated at 37°C and 140 rpm in Tryptone Soja Broth (TSB) to an optical density of 0.8 (OD _650nm_) and cells were stored in Luria broth (LB) media with additional 20% glycerol at– 80°C until usage. Additionally, cells were plated on LB agar plates and incubated at 37°C for 4 days, but no different morphotypes could be detected. To generate strain specific morphotypes, different nutritional limitation conditions were used as described previously [7] with the following modifications. In brief, 10 μl of the strain K96243 and strain E8 glycerol stocks were used for the inoculation of i) 3 ml LB media, ii) 3 ml LB media with 5% glycerol, iii) 3 ml Dulbecco’s Modified Eagle’s Medium (DMEM), iv) 3 ml DMEM media with 5% glycerol, v) 3 ml RPMI-1640 (supplemented with 15 mM citrate) media and vi) 3 ml RPMI-1640 (supplemented with 15 mM citrate) media with 5% glycerol. The cells were aerobically cultured at 37°C under static conditions for 24 days. Afterwards, serial dilutions were plated onto Ashdown agar, incubated at 37°C in air for 4 days and the different colony morphotypes were photographically documented and used for the generation of further morphotype stocks and stored at -80°C. To confirm their stability after storage at -80°C, all morphotypes were plated again on Ashdown agar and incubated for 4 days at 37°C in air, but no further morphotype switching was observed. Three colonies of every morphotype were picked and used as biological replicates in further growth experiments.

### Animal infection experiments and isolation of colony morphotypes after *in vivo* infections

For *in vivo* experiments, female 8 to 12-week-old BALB/c mice were purchased from Charles River (Germany). BALB/c mice were housed under specific-pathogen-free conditions. All mice received 100 colony forming units (CFU) of *B*. *pseudomallei* strain K96243 colony morphotypes intranasally and were monitored daily after infection. After 72 hours, mice were sacrificed and the lungs were homogenized and plated onto Ashdown agar plates in appropriate dilutions. Occurring morphotypes were photographically documented and their colonies were stocked in LB with 20% glycerol at -80°C until usage. Also their stability after storage was confirmed as described above.

### Cell line experiments and isolation of colony morphotypes after *in vitro* infections

For *in vitro* experiments, we used the murine macrophage cell line RAW 264.7 purchased from the American Type Culture Collection (ATCC) (Rockville, MD). Briefly, RAW 264.7 cells were seeded in six-well plates (5 x 10^5^ cells/well) and grown for 24 hours in DMEM + 10% fetal calf serum (FCS) before starting the infection experiments. Cells were infected at a multiplicity of infection of ~5 of all six isolated *B*. *pseudomallei* morphotypes from strain K96243 for 30 min, washed twice with phosphate buffered saline (PBS), then DMEM medium + 10% FCS containing 250 μg/ml kanamycin (Km) was added for 6 hours and finally the cells were further incubated for 18 h with DMEM medium + 10% FCS and 125 μg/ml Km. After a total of 24 hours, cells were washed three times with PBS before lysis with 1 ml *Aqua destillata* at 37°C for 15 minutes. Serial dilutions were spread plated onto Ashdown agar, incubated at 37°C in air for 4 days and occurring colony morphotypes were photographically documented and used for the generation of further *in vitro* morphotype stocks and stored at -80°C. Their stability after storage was confirmed as described above.

### Growth conditions in liquid culture

All morphotypes (obtained from nutrients starvation, *in vivo* and *in vitro* experiments) were spread on Ashdown agar and cultivated at 37°C for 4 days. Then, relevant colonies were scraped from the plates and diluted in 700 μl PBS to an OD _650nm_ of about 10. These dilutions were used for the inoculation of 50 ml RPMI-1640 media containing 15 mM citrate to an OD_650_ of 0.05. All samples were cultured at 37°C and with vigorous agitation for 60 hours. During and after the cultivation an aliquot of the culture was plated on Ashdown agar as described above to confirm the colony morphotype. Cultivations of every morphotype and every condition were carried out as triplicates.

### Sampling and ^1^H-NMR measurements of extracellular samples

2 ml of cell free culture medium were taken at 5.5, 8.25, 11.5, 16.5, 30.5 and 60 hours by sterile filtration and directly frozen until measurement. ^1^H-NMR analysis was carried out as described previously [25]. In brief, 400 μl of the sample was mixed with 200 μl of a sodium hydrogen phosphate buffer (0.2 M, pH 7.0) to avoid chemical shifts due to pH, which was made up with 50% D_2_O. The buffer also contained 1 mM trimethylsilyl propanoic acid (TSP) which was used for quantification and also as a reference signal at 0.0 ppm. To obtain NMR spectra a 1D-NOESY pulse sequence was used with 64 FID scans with 600.27 MHz at a temperature of 310 K using a Bruker AVANCE-II 600 NMR spectrometer operated by TOPSPIN 3.1 software (both from Bruker Biospin).

### Data analysis

For qualitative and quantitative data analysis we used AMIX (Bruker Biospin, version 3.9.14). We used the AMIX Underground Removal Tool on obtained NMR-spectra to correct the baseline. Thereby we used the following parameters: left border region 20 ppm and right border region -20 ppm and a filter width of 10 Hz. The region of noise, used for final baseline correction was between 5.5 ppm and 5.6 ppm. In some cases noise or unknown signals appeared in regions of integrated metabolites after these were consumed completely during cultivation. If these signals were different from the signals in our database they were regarded as false positive. These false positive signals were replaced with an adequate integral of the noise region mentioned. Absolute quantification was performed as previously described [25]. In brief, a signal of the metabolite, either a complete signal or a proportion, was chosen manually and integrated. The area was further normalized on the area of the internal standard TSP and on the corresponding amount of protons and the sample volume.

### Statistics and visualization

Microsoft Excel 2007 was used for all final calculations and for generation of tables. The software PAST was used for the generation of principle component analysis (PCA) [26]. For that matter the calculated concentrations of amino acids were mean centered and autoscaled before being applied to the PCA [27]. Single values from all K96243 morphotypes and *ex vivo* isolates were grouped by the time point of sampling. For statistical comparison of extracellular metabolite data and growth we performed the two-way ANOVA provided by Prism (version 6.01; GraphPad Software). Multiple comparisons were corrected by applying the Holm-Šidák approach and the level on confidence (alpha) was set to 0.01. Bar-charts and XY-plots were also done using PRISM software. Heatmaps of extracellular amino acids were created using MeV v4.8.1 [28] with the following settings for hierarchical clustering: optimized gene leaf order, euclidean distance metric and average linkage method.

## Results

### Induction of colony morphotypes and characterization of their growth in supplemented RPMI medium

**Fig 1:**
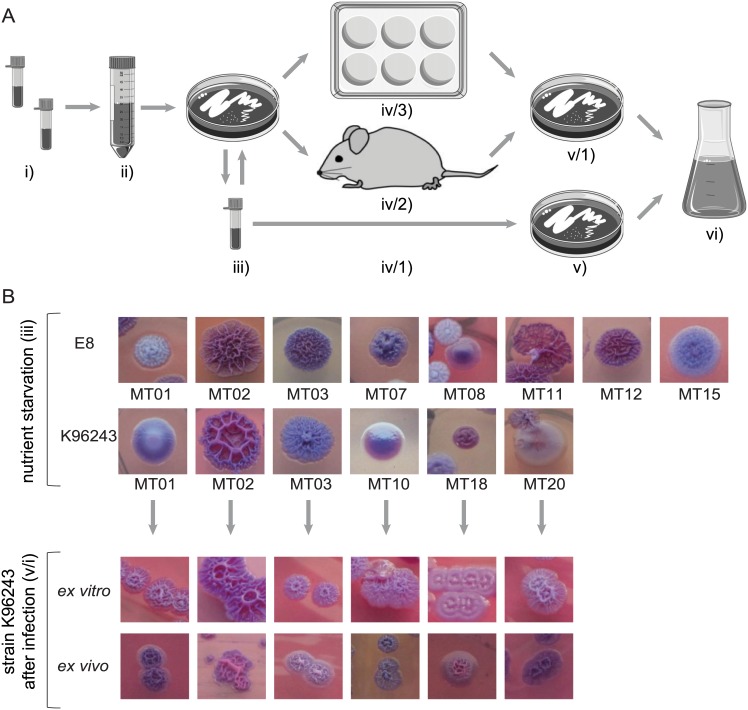
Experimental setup and growth of diverse induced *B*. *pseudomallei* colony morphotypes. **A)** i) Strains E8 and K96243 were taken from glycerol stocks and ii) different media (see Materials and Methods) were inoculated with them for 24 days. iii) Colony morphotypes were identified after plating on Ashdown agar, stored at -80°C and plated again on Ashdown agar to confirm colony morphotype stability for further experiments. iv/1) Glycerol stocks of 14 colony morphotypes from both strains were plated on Ashdown agar (v) and used for inoculation of modified RPMI medium (vi). Obtained morphotypes of strain K96243 were further used in (iv/2) *in vivo* infection experiments with BALB/c mice or (iv/3) for *in vitro* infection experiments in macrophages RAW267.4. v/1) Colony morphotypes after *in vivo* and *in vitro* experiments were identified after plating on Ashdown agar, stored at -80°C and used for inoculation of modified RPMI medium (vi). **B)** Bacteria were plated after (i) nutrient limitation for 28 days (ii) or after (iv/3) *in vitro* and (iv/2) *in vivo* infection, respectively, on Ashdown agar.

We were able to identify 6 morphotypes of the K96243 strain and 8 morphotypes of the E8 strain by plating on Ashdown agar after 24 days of starvation in 6 different media (Fig 1A(iii)). Both strains were able to form rough and smooth colonies with variations in color and inner and outer shape (Fig 1B). We found very similar morphotypes in both strains (E8 MT01 and K96243 MT03; and E8 MT08 and K96243 MT10) but also unique morphologies (E8 MT07, E8 MT12 and K96243 MT20). We used these 14 morphotypes to elucidate differences in uptake and secretion of diverse nutrients and metabolites (Fig 1A(vi)).

**Fig 2:**
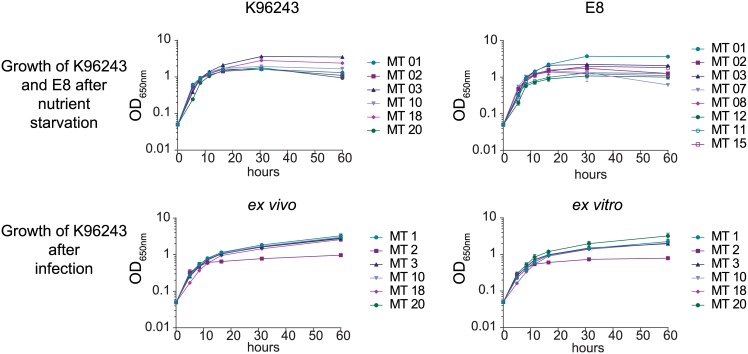
Experimental setup and growth of diverse induced *B*. *pseudomallei* colony morphotypes. Growth curves of selected morphotypes of *B*. *pseudomallei* strains E8 and K96243. Bacteria were grown in modified RPMI medium for 60 h after nutrient limitation conditions and after in vivo infection and in vitro infection. Data are shown as mean values ± SD of triplicate samples.

When we cultivated the morphotypes in modified RPMI for 60 h, no lag phase was detected (Fig 2). After 5 hours the midexponential growth phase was reached and the optical densities of morphotypes differed significantly. After 8.25 hours cells entered the transition phase and subsequently after 30 hours the stationary phase. K9 morphotypes reached maximum optical densities between 1.62 (K96243 MT01) and 3.64 (K96243 MT03) whereas E8 derived morphotypes showed a higher variation between 1.08 (E8 MT12) and 3.73 (E8 MT01). After 60 hours of cultivation we observed cell aggregation in some morphotypes cultures and in accordance to that the optical density, especially in the culture of E8 MT07 and K96243 MT20, dropped slightly.

#### Gluconate production is a common metabolic principle among the colony morphotypes

By using ^1^H-NMR spectroscopy we were able to monitor the concentration of most metabolites, which were present in the initial medium and during the cultivation. This includes mainly amino acids, organic acids, and carbohydrates. An example of a timeline of ^1^H-NMR spectra is given in the S1 Fig. Since carbohydrates are often the main carbon source provided, their consumption is of special interest.

**Fig 3:**
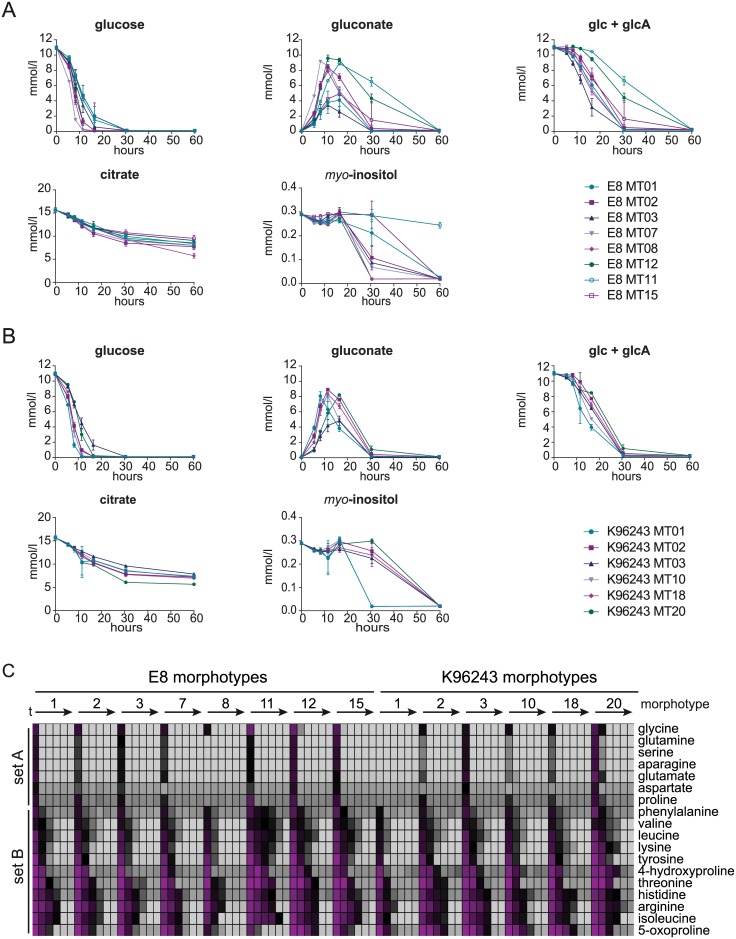
Utilization of extracellular carbon sources and amino acids in *B*. *pseudomallei* starvation induced colony morphotypes. Concentrations (mM) of diverse carbon sources are shown for **A)** E8 and **B)** K96243 starvation induced morphotypes during the cultivation in RPMI medium. Data are shown as mean values ± SD of triplicate samples. **C)** Extracellular amino acid concentrations in starvation induced *B*. *pseudomallei* morphotype cultures during growth are visualized in a color coded chart. The initial concentration in the culture medium was set to 100% and colored magenta whereas 10% is the lower limit and was colored light grey. Displayed are averages of triplicate growth experiments (SD in S1 File, Table A). Arrows indicate the time line of cultivation from 5 h to 60 h for each morphotype.

The initial glucose concentration of 10.96 mM decreased in all cultures but to various extents depending on the morphotype (Fig 3A/3B). It decreased the fastest for E8 MT07 and K96243 MT01 within the first 8.5 h (exponential phase). The time point of complete glucose depletion was also highly diverse between the morphotypes, varying between 11 and 30 hours of cultivation. Simultaneously to extracellular glucose depletion we detected rising concentrations of gluconate in the medium (Fig 3A/3B). Also similar to glucose consumption, gluconate production was highly variable between the morphotypes. Maximum amounts of gluconate were found especially in morphotypes of strain E8 at various time points. After 8.25 h of cultivation 9.17 mM gluconate was detectable in the culture of E8 MT07, whereas in cultures of MT12 and MT11 9.58 mM and 8.29 mM were measured after 11 h and 16 h respectively. The amounts correspond to 81%, - 88% relatively to the initial concentration of glucose. Other morphotypes were found to have comparably low maximal gluconate concentrations which corresponded to only 31% - 37% of the total glucose concentration. After transition phase, when the gluconate concentration peaked, it dropped again until gluconate was abolished between 30 h and 60 h. We assumed that glucose is the precursor of gluconate production and examined the consumption of the combined concentration of glucose and gluconate (Fig 3A/3B). In fact, during the first 5 hours (until midexponential phase) no significant decrease in the combined concentration was observed. The time point of a significant depletion of glucose and gluconate varied between the morphotypes in a wide range from the late exponential phase to the stationary phase. At the 16 hour time point (transition phase) the combined concentration of glucose and gluconate varied the most between 28% and 94% of the initial available concentration.

### Citric acid is co-metabolized with other nutrients in contrast to myo-inositol

Beside glucose, citrate and *myo*-inositol are available in the modified RPMI medium and serve as carbon sources. Notably, citrate was present after supplementation in a concentration of 15.65 mM whereas *myo*-inositol was rather low concentrated at 0.29 mM. All morphotypes showed citrate uptake with some variation over time (Fig 3A/3B). Unlike glucose or amino acids, citrate was not completely depleted after 60 h of cultivation but the uptake was strongly reduced after 30 h for most morphotypes. The decrease in concentration of citrate within the first 30 h of cultivation was measured between 4.91 mM and 9.55 mM and only between 0.65 mM and 3.49 mM during the second 30 hours. A significant uptake of *myo*-inositol was first measured 16 h or 30 h after inoculation, depending on the morphotype (Fig 3A/3B). K96243 MT01 and K96243 MT10 and E8 MT08 and E8 MT12 showed the fastest *myo*-inositol uptake. E8 MT11 however showed only very little uptake of *myo*-inositol.

### Amino acids are taken up with different rates and with variation between the colony morphotypes

Based on the decline in concentration, amino acids were classified in two sets (Fig 3C). Set A consists of the amino acids glutamine (gln), glutamate (glu), aspartate (asp), asparagine (asn), serine (ser), glycine (gly) and proline (pro) and showed a decline in concentration below detection limit within the exponential growth phase. In the culture of the morphotypes K9 MT01 and E8 MT08 the uptake of most set A amino acids was completed even before reaching midexponential phase. Set B includes aromatic amino acids (tyrosine (tyr), phenylalanine (phe) and histidine (his)), branched chain amino acids (isoleucine (ile), leucine (leu) and valine (val)), positive charged amino acids (lysine (lys) and arginine (arg)), non-proteinogenic (4-hydroxyproline, 5-oxoproline) and threonine (thr). Amino acids of set B were consumed slower during growth and for some morphotypes almost no uptake was observed until midexponential phase (5 hours). This applies especially to thr, of which more than 96% of the initial amount was still being available in all morphotypes cultures after 5 h. Also only little amounts of arg, tyr, 4-hydroxyproline and 5-oxoproline are taken up in that time. In the late transition phase the diversity between the uptake profiles was relatively high, since some morphotypes took up amino acids of set B more efficiently than others. Interestingly, E8 MT11 showed the fastest uptake of the non-proteinogenic amino acid 5-oxoproline, whereas being rather slow in the uptake of other amino acids of set B. After 30 hours only two morphotype cultures still contained amino acids in measurable amounts (ile; val; leu in the culture of E8 MT11 and arg in the culture of K96243 MT20). However, at the end of cultivation a complete uptake of amino acids was observed for all morphotypes.

### Intermediates of branched chain amino acid metabolism are secreted by some colony morphotypes

**Fig 4:**
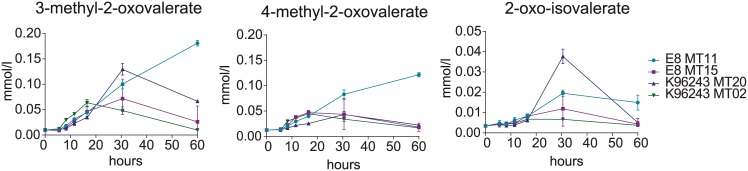
Secretion of BCAA metabolism intermediates. Absolute extracellular concentrations of secreted BCAA-pathway metabolites are displayed. The two most prominent morphotypes of K96243 and E8 after nutrient limitation are displayed. Data are shown as mean values ± SD of triplicate samples.

The first step in branched chain amino acid (BCAA) degradation is the deamination via IlvE (branched chain amino acid aminotransferase). We found the corresponding α-keto-acids (leu→ 4-methyl-2-oxovalerate; ile→ 3-methyl-2-oxovalerate; val→ 2-oxoisovalerate) to be secreted into the medium by several morphotypes in minor but distinct amounts (Fig 4). Overall, K96243 MT20, E8 MT11 and E8 MT15 secreted the highest amounts. Notably for E8 MT11 no reuptake of these compounds was observed, whereas the other morphotypes consumed the secreted metabolites again. The reuptake for K96243 MT20 and E8 MT15 started after 30 hours of growth, when branched chain amino acids were mostly depleted, yet for E8 MT11 the uptake of branched chain amino acids was still in progress. Therefore the reuptake of α-keto-acids may be connected to the depletion of corresponding amino acids.

Isovalerate, which might be a degradation product of leucine, was solely found in the culture medium of two E8 morphotypes (MT03 and MT15) (S2 Fig). Secretion of isovalerate started in the transition phase and no reuptake for this substance was observed in both cultures.

Several other signals were appearing in the spectroscopic data and point to the secretion of other organic compounds into the medium especially, when cells enter the stationary phase. Unfortunately we were not able to identify most of these metabolites (detailed signal pattern are summarized in S1 File, Table A). However, even if the signals are unknown, they can be used for a discrimination of morphotypes by their secretion pattern (S2 Fig).

### *In vivo* and *in vitro* infection induced a switch to a single colony morphotype and similar growth in RPMI medium

To elucidate the effect of infection on morphotype stability and metabolic activity we performed infection experiments in mice (Fig 1iv/2) and murine macrophage cell cultures (Fig 1iv/3) and isolated bacteria out of the lung and the intracellular compartment of macrophages. Post *in vivo* and *in vitro* infection (p.i.), the isolated K96243 morphotypes showed a homogenous rough colony, which was similar to the K9 MT03/MT02 morphotype prior to infection (a.i.) (Fig 1B). These isolates were cultivated again in liquid modified RPMI medium for 60 hours and samples to investigate the metabolic footprint were taken. We found that, in contrast to a stationary phase, a second lower growth rate was established after the exponential phase and remained until the end of the experiment (Fig 2). Most isolates of *in vitro* infections were growing within 60 hours to similar optical densities between 1.97 and 2.24, whereas MT20 grew up to 3.21 and MT02 only reached 0.79. A slightly higher growth was determined for isolates after *in vivo* infection which grew to optical densities between 2.57 and 3.22 with MT02 being an exception with a maximum of only 0.96.

### Usage of carbon sources in the cultivation of *B*. *pseudomallei* colony morphotypes after infection

**Fig 5:**
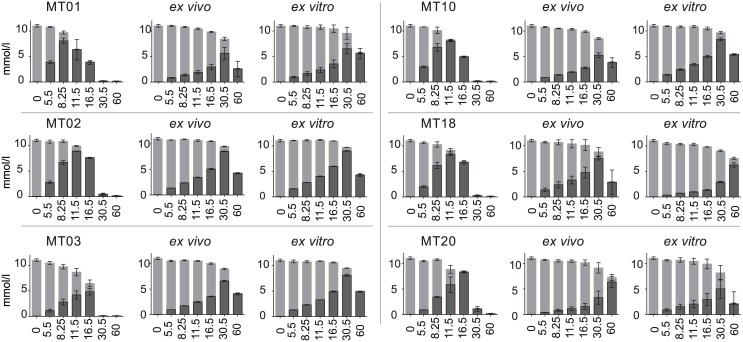
Comparison of extracellular glucose and gluconate concentrations in starvation and infection induced colony morphotype cultures. Absolute extracellular concentrations of glucose (light grey) and gluconate (dark grey) in starvation and infection (*in vivo* and *in vitro*) induced K96243 morphotype cultures over time are displayed. Data are shown as mean values ± SD of triplicate samples.

After *in vitro* and *in vivo* infection the isolates were grown again in modified RPMI medium for 60 h. The glucose concentration decreased significantly within the first 8.5 h (exponential phase) in all isolate cultures but slower compared to the a.i. cultivation (Fig 5). Whereas after cultivation for 16 hours pre infection glucose was depleted in the culture media, the lowest glucose concentration was measured in the K96243 MT02 (*ex vitro*) culture with still 4.97 mM. Other morphotypes were found with even higher extracellular glucose concentrations of up to 8.58 mM. Consequently, we detected gluconate in morphotype cultivations p.i. in slower rising concentrations (Fig 5). The maximum extracellular gluconate concentration was measured for most morphotypes p.i. after 30 h of cultivation and reached up to 9.00 mM. At 60 h of cultivation the concentration of gluconate decreased in all morphotype cultures p.i. except for MT18 *ex vitro* and MT20 *ex vivo*. A significant reduced combined concentration of glucose and gluconate was first measured 30.5 h after inoculation for all isolates except MT02 *in vitro* and MT18 *in vitro* (lower but not significant amount) (Fig 5). Whereas prior to infection most of the glucose and gluconate was taken up during the transition phase, in the cultivation p.i. the majority was consumed in the slow growth phase between 30 h and 60 h. In this time period the variation in glucose and gluconate consumption among the morphotypes increased significantly. Other carbon sources were used less after infection. The total amount of consumed citrate was reduced from 8.58 ± 0.74 mM in average pre infection to 3.52 ± 0.42 mM and 3.90 ± 0.65 mM post *in vivo* and *in vitro* infection respectively (S3 Fig). Notably the vast majority of citrate was taken up after 16 h of growth. The highest variation in extracellular citrate concentrations was observed at the very end of the experiment. *Myo*-inositol remained at the initial level for most morphotypes and no uptake was observed (S3 Fig).

### The infection process led to similar amino acid uptake profiles among the colony morphotypes

**Fig 6:**
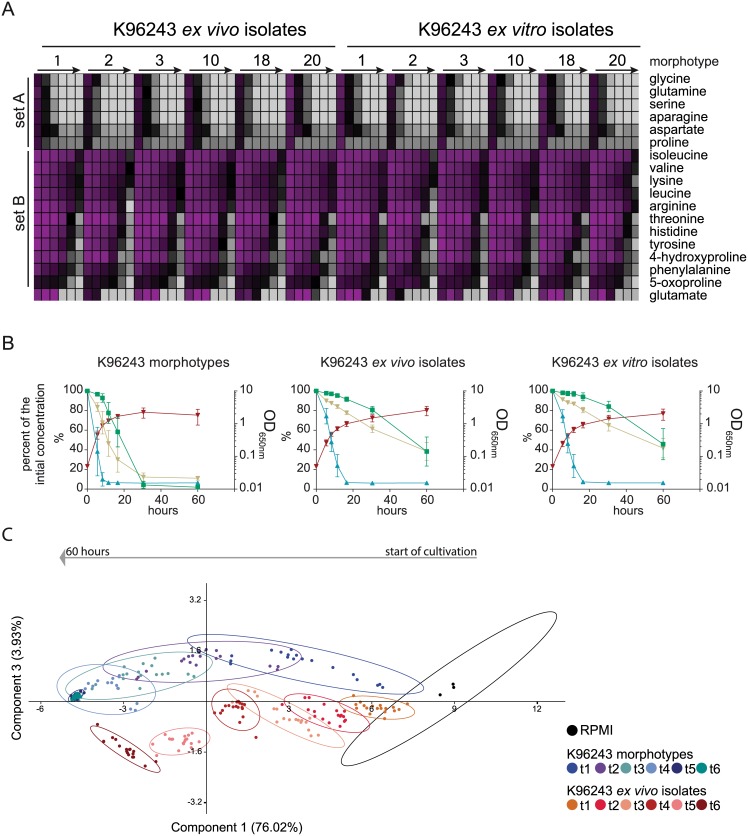
Consumption of extracellular amino acids in *B*. *pseudomallei* infection induced colony morphotypes. **A)** Extracellular amino acid concentrations during the cultivation of K96243 morphotypes after *in vitro* (Fig 1(iv/3)) and *in vivo* (Fig 1(iv/2)) infection conditions are visualized in a color coded chart. The initial concentration in the culture medium was set to 100% and colored magenta whereas 10% is the lower limit and was colored light grey. Displayed are averages of triplicate growth experiments (SD in S1 File, Tables B and C). Arrows indicate the time line of cultivation from 5 h to 60 h for each morphotype. **B)** The combined value of optical densities (OD_650nm_) of all K96243 morphotypes are displayed in red lines. Nutrition sets contain either glc+glcA (green), amino acids of set A (asp, asn, glu, gln, ser, pro, gly) (blue) or amino acids of set B (his, tyr, thr, phe, ile, val, leu, arg, lys, 5-oxoproline, 4-hydroxyproline) (brown). Percentage values of nutrition sets in K96243 morphotype cultures before or after *in vivo* and *in vitro* infection were calculated by summing up the average amount of the metabolites within a set and comparison to the initial value in the medium prior to cultivation (≙100%). Data are shown as mean values ± SD. **C)** The calculated amino acid concentrations of K96243 morphotypes after (Fig 1(iv/1)) nutrient limitation and (Fig 1(iv/2)) *ex vivo* isolates were mean centered and autoscaled and applied to principle component analysis. Clusters were generated by adding each sample, of all 6 morphotypes and 6 *ex vivo* isolates respectively, of the same time point to one group (t_1_-t_6_). The arrow above the plot indicates the time course. Groups are indicated by the same color and statistically clustered by an ellipse with a confidence interval of 90%. Displayed is component 1 versus component 3 with their corresponding proportion of variation.

The amino acid uptake profile of cultivated morphotypes p.i. showed similarities to the morphotypes a.i. with regard to the classification of amino acids. We found a set of amino acids with a fast uptake profile (set A: gly, ser, pro, gln, asp, asn) and a slow uptake (set B: arg, leu, ile, val, his, lys, thr, tyr, phe, 5-oxoproline and 4-hydroxyproline) (Fig 6A). Additionally, glutamate showed rising concentrations in the extracellular space of some morphotypes indicating a secretion of this amino acid. Subsequently, it was consumed in a fast manner. In all morphotype cultures p.i. (*in vitro* and *in vivo)* amino acids of set A are still present 5 h after inoculation, which was not the case before infection. Some amino acids of set B, especially branched chain amino acids, were taken up to a much lesser extend and were not depleted after 60 h of cultivation. For threonine no significant uptake could be measured until 30 h of cultivation for any morphotype. For isoleucine and arginine no uptake between mid-exponential phase and 16 h, 30 h or 60 h depending on the morphotype was observed. In general the data show, that usage of both sets was slower after infection, compared to the cultivation prior to infection (Fig 6B). All K96243 morphotypes showed p.i. (*in vivo* and *in vitro*) a more focused use of set A amino acids during the exponential phase. After 16.5 hours approximately 25% of set B amino acids were used in comparison to about 75% prior to infection in the same time (Fig 6B). In the later growth phase, the content of set B amino acids was decreased to 60% in the p.i. cultivation but completely consumed in the a.i. cultivation. Furthermore, the morphotypes p.i. showed very small variation in the usage of amino acids. This can be also shown when we apply the amino acid concentrations to principle component analysis (Fig 6C). The PCA plot of PC1 versus PC3 shows that K96243 morphotypes a.i. form wide spread and overlapping groups which correlate with diverse amino acid uptake profiles compared to morphotypes p.i., which group in smaller clusters and more distinct. The PCA plot of PC1 versus PC2 shows a orthogonal structure caused by the successive uptake of the two amino acid sets as indicated by the loading plot of PC2 (S4 Fig). Even if the morphotypes p.i. are very similar, especially MT02 showed differences compared to the other morphotypes. After *in vivo* and *in vitro* infection, the uptake of glu, gln, leu, ile and arg was faster in MT02 than in other isolates. In fact MT02 was the only isolate, which consumed arg completely within 60 h. Additionally a faster uptake was also seen for his, asp, asn, and thr only after *in vitro* infections. This is indeed surprising since MT02 showed reduced growth compared to the other morphotypes with regard to the optical density.

### Secretion of intermediates of branched chain amino acid metabolism after infection

**Fig 7:**
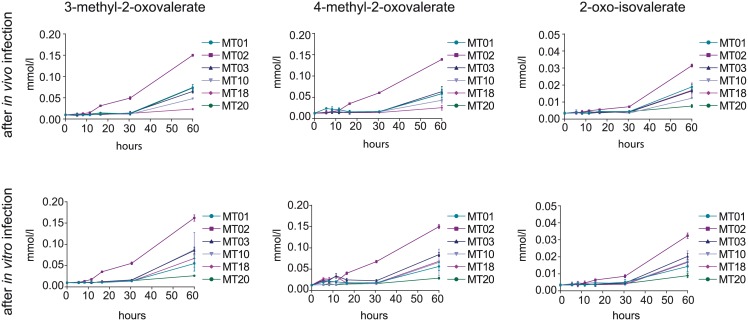
Secretion of BCAA metabolism intermediates by infection induced colony morphotypes. Secreted amounts of α-keto-acids by K96243 isolates after *in vivo* (Fig 1(iv/2)) and *in vitro* (Fig 1(iv/3)) infection are shown. Data are shown as mean values ± SD of triplicate samples.

Prior to infection K96243 MT20 was found to produce the majority of α-keto-acids of all K96243 morphotypes and an additional reuptake of these compounds was observed over time. However, after infection these metabolites were minimally secreted in any morphotype culture within the first 30 h of cultivation. Isolates of K96243 MT02 were found to produce the majority of α-keto-acids starting at 11.5 h or 16 h respectively (Fig 7). Also no reuptake of secreted metabolites was measured. Similar to growth, amino acid uptake and carbon assimilation isolates of *in vivo* and *in vitro* infections show rather similar secretion patterns during cultivation (S4 Fig).

## Discussion

In our study, we observed major qualitative similarities in metabolic behaviour of the investigated *B*. *pseudomallei* morphotypes like gluconate production and amino acid uptake. However, we also observed various differences in growth, uptake rates and metabolite secretion patterns supporting the idea, that beside the similarity, morphotypes exhibit different regulations on a metabolic level. Interestingly, these variations were strongly reduced, after the morphotypes were isolated from the lung of infected mice or from the intracellular environment of murine macrophages.

Extracellular gluconate production was described before for other proteobacteria like *Pseudomonas fluorescence*, *Pseudomonas cepacia*, and *B*. *cepacia* and is thought to be connected to the mineral phosphate solubilizing ability of gluconate [29–31]. In a metabolic profiling study, it was shown, that clinical *P*. *aeroginosa* isolates from various cystic fibrosis patients do secrete gluconate, and that a small non-coding RNA (CrcZ) regulates gluconate production [32]. Extracellular gluconate is produced by a periplasmatic glucose dehydrogenase (Gdh) and can be further converted by a gluconate dehydrogenase (Gad) into 2-keto-gluconate. Gluconate or 2-keto-gluconate can then be taken up by the cells and further metabolized by either the Entner-Doudoroff-pathway (ED-pathway) or by the pentose-phosphate-pathway (PP-pathway). Consistently, a study investigating intracellular carbon fluxes for various bacteria (among these *P*. *fluorescence* and *P*. *putida*) showed, that the ED-pathway is the main pathway for carbon catabolism in many bacteria [33]. Surprisingly, neither *gdh*, nor *gad* is present in the genome of *B*.*pseudomallei* and moreover, we did not detect 2-keto-gluconate in the extracellular space. Therefore, it is still unknown whether gluconate is produced by an unassigned glucose dehydrogenase in the periplasm or by an intracellular glucose dehydrogenase followed by a subsequent secretion into the medium. In the case of *P*. *fluorescence* it was described, that gluconate is taken up when glucose is depleted [33]. We also observed gluconate uptake when the extracellular glucose level reached a minimum. Yet with regard to the extracellular gluconate concentration we found two variations. Some morphotypes reached high extracellular gluconate concentrations (71%-88% of the provided glucose) and some cultures contained less gluconate (31%-45%). Either gluconate production is lower in some morphotypes and glucose is taken up directly or the gluconate uptake rate is higher in these morphotypes. If direct glucose uptake is enhanced in these morphotypes they do not show a significant difference in glucose depletion compared to the other morphotypes. Therefore, we would rather suggest that gluconate uptake is elevated in these morphotypes. We found, that low extracellular gluconate concentrations correlated with higher final optical densities, suggesting a faster gluconate uptake favors growth. In general, these data imply that typical gluconate catabolizing pathways as the ED-pathway or the PP-pathway are the preferred pathways for carbohydrate catabolism instead of the Embden-Meyerhof-Parnas-pathway in *B*. *pseudomallei*.

After being isolated from the intracellular environment, we found that the uptake of glucose and gluconate was strongly delayed and very similar among all isolates, which consumed mainly certain amino acids during exponential growth. For many bacteria a regulative process called carbon catabolite repression (CCR) is known, which regulates the hierarchy of carbon assimilation and might also play a role in *B*. *pseudomallei*. As mentioned above there are similarities between the genus *Burkholderia* and the close related bacterial family of Pseudomonads. *Pseudomonas* species exhibit a CCR-system that is different from model organisms like *Escherichia coli* and *Bacillus subtilis*. Contrary to these species, Pseudomonads prefer organic acids and amino acids as carbon sources rather than glucose or other sugar derivatives including gluconate [34,35]. Not only the preference for the carbon source is different, but rather the whole CCR-system. Whereas a phosphotransferase-system senses the availability of preferred carbon sources in *E*. *coli* and *B*. *subtilis*, in *Pseudomonas* sp. a protein called Crc acts as a global regulator controlled by CrbA, CbrB and the above mentioned small non coding RNA CrcZ [34,36]. Crc does not only distinguish between amino acids and sugars it is also able to establish a hierarchy of amino acid uptake [37]. Unfortunately, very little is known about the process of carbon catabolite repression in *Burkholderia* species. For *B*. *cepacia* the expression of a gene (*alkB*) involved in alkane degradation as a carbon source seems to be also regulated by catabolite repression [38]. A genome wide search in *B*. *pseudomallei* for CrcZ, CbrA and CbrB was without result, but we found a homolog to Crc from *P*. *aeruginosa* with 41.9% identity to BPSL0191 in *B*. *pseudomallei*. Therefore a similar CCR as described for *P*. *aeruginosa* is possible but still speculative in *Burkholderia* species. Even though the molecular mechanisms of carbon catabolite repression in *Burkholderia sp*. are unclear, our data indicate that carbon source uptake might be regulated in *B*. *pseudomallei* and that certain amino acids (gln, glu, asn, asp, ser, gly, pro) are the preferred carbon sources during exponential growth, especially after infection. Some amino acids of set B (thr, arg, and ile) showed not only a slower uptake over time but rather a delayed uptake, which also points to a hierarchical uptake of amino acids. Another indication towards carbon catabolite repression is the uptake of *myo*-inositol. It only occurred when glucose and gluconate were depleted, in opposite to citrate which was consumed mostly with other nutrients still being present.

A possible explanation for the distinct regulation of nutrition uptake after infection might be an adaptation to the environment during infection. After *in vivo* infection, bacteria were isolated from the lung and not differentiated between intra- and extracellular bacteria, whereas in our *in vitro* approach, bacteria were isolated solely from the intracellular compartment. Interestingly, there was almost no difference in the metabolic behavior between *ex vivo* and *ex vitro* isolates suggesting, that the trigger for morphotype switches and changes in metabolic behavior is present in both infection models. We therefore favor the idea, that the trigger that induces morphotype switching is an intracellular signal. Especially intracellular pathogens must have the ability to use nutrients provided by the host cell to satisfy their own needs for replication, since the host-cell cytosol is not a suitable growth medium for bacteria in general [39]. Therefore intracellular pathogens like *Francisella tularensis* and *Legionella pneumophila* have developed strategies to manipulate cellular host processes like autophagy or proteasomal degradation to elevate the amount of free amino acids [40,41]. So far, such mechanisms are not known for *B*. *pseudomallei*, but like the two former mentioned bacteria, it also shows defects in intracellular replication in a mouse model when the stringent response, an amino acid sensing system, is defect [20]. The slower uptake of certain amino acids after infection could be explained, if, due to the low intracellular abundance in the host, these metabolites have to be synthesized and therefore uptake systems for these metabolites might be downregulated.

Biosynthesis of BCAAs is essential for *B*. *pseudomallei* to establish long time persistence and the demand cannot be satisfied by host cell derived BCAAs [42]. Indeed, our data suggest that *B*. *pseudomallei* is rather used to low amounts of BCAAs since we found that some morphotypes secreted 3-methyl-2-oxovalerate, 4-methyl-2-oxovalerate and 2-oxoisovalerate during cultivation. Interestingly, the secretion of isovalerate by only two E8 morphotypes indicates morphotype specific metabolic aspects of leucine degradation, whereby the exact pathway remains unclear [43]. A secretion of degradation products of branched chain amino acids has been described previously for two *S*. *aureus* strains that were grown in RPMI medium [25]. These metabolites are either substrates of branched-chain amino acid aminotransferase (IlvE) during leucine, isoleucine and valine synthesis, respectively, or products of IlvE, when BCAAs are deaminated during degradation. We also could observe a reuptake of these metabolites in stationary phase, indicating that during exponential growth BCAAs are consumed in excess and cannot be used for biosynthesis or catabolism. Similar to auxothrophic mutants for BCAA-biosynthesis mutants in other pathways like purine synthesis, histidine synthesis and *p*-aminobenzoic acid synthesis showed attenuated virulence and growth defects in a mouse model [44]. This confirms the lack of specific nutrients inside the host. Such mutants in biosynthesis pathways of essential metabolites are of clinical importance because of their display of potential candidates for vaccination [45].

However, the metabolic adaption and morphotype switch we present in our study is caused by acute infection in a mouse pneumonia model or in a murine macrophage cell line and might only represent a short episode of adaptation. Obligate intracellular bacteria like *Buchnera*, *Wigglesworthia* and *Blochmannia*, which are highly adapted to the host, show extensive gene reductions of housekeeping genes and thereby strong dependences of host nutrient supply [16]. And indeed, a recent study shows that after long-term (12 years) persistence biosynthetic pathways for amino acids are lost due to genome reduction in *B*. *pseudomallei* which indicates usage of host provided amino acids or redundant pathways [46].

Further research is needed to investigate i) the biosynthesis pathways, which are required for *B*. *pseudomallei* to establish an infection and ii) the intracellular conditions in the host cell, which allow microbial replication. Additionally to metabolic footprints, the intracellular metabolome, the fingerprint, should be examined to identify metabolic differences between *B*. *pseudomallei* morphotypes and particularly to uncover the metabolic state of the morphotype after infection. We would favor a combination of biosynthesis pathway mutants and metabolome approaches to uncover the dependence of the microbial metabolism on host derived metabolites.

Overall our metabolic footprint study provides for the first time insights into the so far unknown metabolic characteristics of *B*. *pseudomallei* morphology variants. The finding of gluconate production points out, that metabolome approaches are needed to describe the metabolism of an organism, despite the availability of genomic data, since no gluconate production enzymes were assigned so far for *B*. *pseudomallei*. We identified a synchronization effect in colony morphology and metabolism due to acute infection that might play an important role in the pathogenicity of *B*. *pseudomallei*. Our metabolomic study therefore contributes to the necessary knowledge about a hazardous pathogen and its adaption to the host in the acute phase of melioidosis.

## Supporting Information

S1 Fig Example ^1^H-NMR spectra of a timeline of cultivation samples. A section of ^1^H-NMR spectra of the cultivation samples of E8 MT01 from t_1_—t_6_ is presented. The decrease of the signals for glucose, *myo*-inositol and some amino acids at various time points is visible as well as the increase and further decrease of the gluconate signal.
(EPS)

S2 Fig Color coded chart of extracellular concentrations of all integrated signals in the medium of starvation induced colony morphotypes. Average values (n = 3; SD in S1 File, Table A) of absolute concentrations for identified metabolites and of relative concentrations for unknown metabolites were log_2_ transformed and displayed as the colour code indicates. Arrows indicate the time line of cultivation from 5 h to 60 h for each morphotype and the first column represents initial values in medium. Unknown signals are named according to their signal multiplicity and chemical shift.
(EPS)

S3 Fig Uptake of other carbon sources is reduced in all *B*. *pseudomallei* cultures post infection. Average concentrations (n = 3; ±SD) of **A)** citrate or **B)** *myo*-inositol are shown for all cultivations of all *ex vivo* and *ex vitro* K96243 colony morphotypes.
(EPS)

S4 Fig Principle component analysis of the amino acid uptake. **A)** The calculated amino acid concentrations of K96243 morphotypes after (Fig 1(iv/1)) nutrient limitation and (Fig 1(iv/2)) *ex vivo* isolates were mean centered and autoscaled and applied to principle component analysis. Clusters were generated by adding each sample, of all 6 morphotypes and 6 *ex vivo* isolates respectively, of the same time point to one group (t_1_-t_6_). Groups are indicated by the same color and statistically clustered by an ellipse with a confidence interval of 90%. Displayed are component 1 versus component 2 with their corresponding proportion of variation is displayed. **B)** Loading plots of PC1 and PC2.
(EPS)

S5 Fig Color coded chart of extracellular concentrations of all integrated signals in the medium of infection induced colony morphotypes. Average values (n = 3; SD in S1 File, Tables B and C) of absolute concentrations for identified metabolites and of relative concentrations for unknown metabolites were log_2_ transformed and displayed as the colour code indicates. Arrows indicate the time line of cultivation from 5 h to 60 h for each morphotype and the first column represents initial values in medium. Unknown signals are named according to their signal multiplicity and chemical shift.
(EPS)

S1 File Metabolome data. A list of all metabolites and unknown signals with the region of integration in the NMR spectrum is provided. The file contains tables with the calculated concentrations of **A)** the cultivation of K96243 and E8 colony morphotypes and the cultivation K96243 colony morphotypes after **B)** *in vivo* and **C)** *in vitro* infection. For all average values the according standard deviation is provided. Table **D)** contains the loadings of the PCA presented in Fig 5C.
(XLSX)
